# Orphan Enzymes in the Mammalian L-Fucose Degradation Pathway

**DOI:** 10.3390/biom16070985

**Published:** 2026-07-04

**Authors:** Apolonia Witecka, Julia Zuzanna Kamińska, Klaudia Ślusarczyk, Jan Jakub Piętka, Mikołaj Witczak, Sebastian Kwiatkowski, Jakub Drożak

**Affiliations:** 1 Department of Metabolic Regulation, Faculty of Biology, University of Warsaw, 1 Miecznikowa Street, 02-096 Warsaw, Poland; aa.witecka@uw.edu.pl (A.W.); jz.kaminska@uw.edu.pl (J.Z.K.); klaudia.slusarczyk@imid.med.pl (K.Ś.); jj.pietka@student.uw.edu.pl (J.J.P.); ma.witczak@student.uw.edu.pl (M.W.); 2 Doctoral School of Translational Medicine, Centre of Postgraduate Medical Education, 99/103 Marymoncka Street, 01-813 Warsaw, Poland

**Keywords:** orphan enzymes, L-fucose, L-galactose, D-arabinose, catabolic pathway, human, mammals

## Abstract

Orphan enzymes are recognized and classified enzymatic activities that lack associated amino acid sequences. Since the term was coined in the mid-2000s, the proportion of orphan enzymes has substantially decreased; however, it is estimated that at least ≈900 enzymatic activities remain devoid of molecular identity to date. The putative mammalian metabolic pathway for L-fucose degradation represents a system that long consisted exclusively of orphan enzymes, with only a few recently “deorphaned” and biochemically characterized. L-Fucose is a unique monosaccharide frequently found in various glycolipids and glycoproteins synthesized by mammalian cells, such as the ABO blood group antigens in humans. While the importance of the biosynthetic pathways for its active form (GDP-L-fucose) is well established in diverse biological processes, the enzymology and physiological role of L-fucose catabolism remain largely enigmatic. In this review, we summarize the current knowledge regarding the enzymological and physiological aspects of L-fucose catabolism in mammals.

## 1. Introduction

### 1.1. Orphan Enzymes

With the deluge of complete genome sequences submitted to public databases over the past twenty years—reaching 6000 unique genomes for eukaryotic species alone as of 2026—it has quickly become evident that a surprisingly high number of well-known and classified enzymes lack any associated amino acid sequences [1,2]. In the mid-2000s, the term “orphan enzymes” was coined to distinguish this group, prompting public calls for community-wide mobilization to identify at least one protein sequence for every biochemically characterized enzyme [2,3]. Since then, the proportion of orphans has indeed decreased from 36% (in 2006) to 16% (in 2025) of all Enzyme Commission numbers (EC numbers) listed in the dedicated database ORENZA https://bioi2.i2bc.paris-saclay.fr/orenza/ (accessed on 11 May 2026) [4]. Nevertheless, it is estimated that at least ≈900 activities remain to be deorphaned. Furthermore, given that not all reported enzyme activities have been assigned EC numbers, the true number of orphans is likely even higher.

Elucidating the molecular identity of orphan enzymes is a significant challenge; however, when successful, it provides a vital bridge between protein function and sequence. This connection is crucial for comparative genomics and frequently offers a profound understanding of enzymatic biochemistry and its (patho)physiological significance. Importantly, the implications of such findings can revitalize long-forgotten research problems or open entirely new areas of study.

For example, research into the molecular identification of an orphan ATP-dependent dehydratase acting on the endogenous damaged form of NAD(P)H (NAD(P)HX) (EC 4.2.1.93), first described in the 1950s [5], led to the discovery of a highly evolutionarily conserved enzymatic system that repairs NAD(P)HX [6]. This serves as a prominent example of so-called metabolite repair enzymes (for review, see [7]). This identification also provided a mechanistic explanation for severe and lethal encephalopathy in children suffering from deficiencies in NAD(P)HX repair enzymes [8].

Similarly, the molecular identification of an orphan actin-specific histidine methyltransferase (EC 2.1.1.85) [9,10] as the SETD3 protein in Metazoa sparked renewed interest in the biochemistry and physiological importance of histidine-specific protein methyltransferases [11,12,13,14,15]. This topic had been largely neglected since pioneering work in the 1960s–1980s (for review, see [16,17]). These and other discoveries [18,19,20] in the field of orphan enzymes underscore the importance of systematic studies in this area to better understand cellular biochemistry.

### 1.2. Orphan Enzymes in L-Fucose Catabolism

Many orphan enzymes catalyze reactions integral to metabolic pathways [21,22]. Early surveys of the KEGG Pathway database revealed that the majority of pathways (≈90%) contain at least one orphan activity [21]. Furthermore, our studies suggest that certain biochemical pathways consist largely of enzyme activities whose molecular identities remain unknown. A primary example is the breakdown of L-fucose in mammals—a putative catabolic pathway observed in the mammalian liver and kidneys that involves a few orphan enzymes.

**Figure 1:**
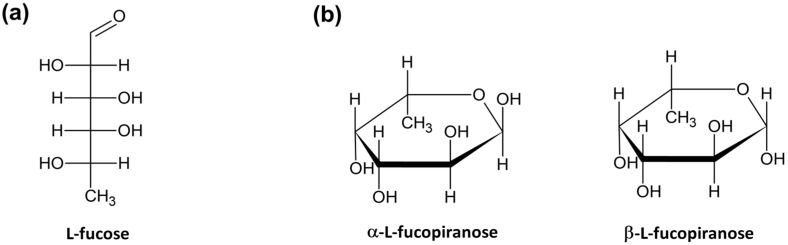
The structure of L-fucose. The monosaccharide is shown in (**a**) the Fisher and (**b**) Haworth projections.

**Figure 2:**
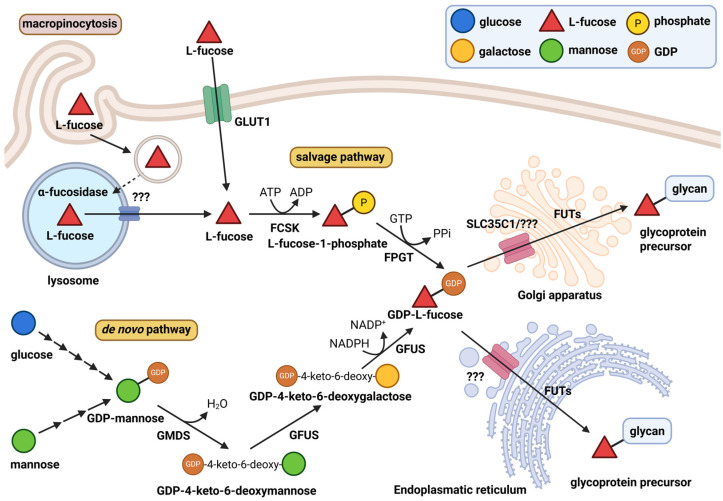
Metabolic pathways of GDP-fucose biosynthesis in mammalian cells. GDP-L-fucose, the activated form of the monosaccharide, is synthesized via two distinct routes: the de novo biosynthesis pathway and the salvage pathway. The de novo pathway initiates with the conversion of glucose or mannose into GDP-mannose. Subsequently, GDP-mannose is dehydrated to GDP-4-keto-6-deoxymannose, which is then epimerized to GDP-4-keto-6-deoxygalactose. This intermediate is reduced to GDP-fucose in an NADPH-dependent reaction (for review, see [23,24]). The salvage pathway involves the uptake of extracellular L-fucose via the GLUT1 transporter and through macropinocytosis. Additionally, L-fucose can be liberated from fucosylated glycans by α-fucosidases during lysosomal degradation [25]. While the lysosomal transporter responsible for releasing free L-fucose into the cytosol remains unidentified, cytosolic L-fucose is phosphorylated to L-fucose-1-phosphate, which serves as a substrate for a guanylyltransferase that replaces the phosphate group with a GDP moiety. The GDP-fucose produced by both pathways is transported into the lumen of the Golgi apparatus by the transporter SLC35C1 and into the endoplasmic reticulum (ER) by an as-yet-unidentified protein [26]. Within these organelles, fucosyltransferases (FUTs) catalyze the incorporation of L-fucose into glycans to form mature glycoproteins (for review, see [23,27]). Additionally, an alternative, currently uncharacterized mechanism has been reported to transport salvage-derived GDP-fucose to the Golgi apparatus independently of SLC35C1 [28]. Abbreviations: GMDS, GDP-mannose 4,6-dehydratase; GFUS, GDP-L-fucose synthase; GLUT1, glucose transporter type 1; FCSK, L-fucose kinase; FPGT, fucose-1-phosphate guanylyltransferase; FUTs, fucosyltransferases; ???, unknown mechanism. Created in BioRender. Drozak, J. (2026) https://BioRender.com/cyg6hvw (accessed on: 18 June 2026).

L-Fucose (6-deoxy-L-galactose) is a unique monosaccharide that occurs frequently in a variety of glycolipids and glycoproteins synthesized by mammalian cells (Figure 1) (for review, see [23]). It is distinguished by its L-configuration and the absence of a hydroxyl group at the C6 position. L-Fucose is present in approximately 7% of mammalian oligosaccharides, occurring either as a terminal modification or within the core structure of glycans. Additionally, this monosaccharide can be directly linked to the serine or threonine residues of proteins [23]. Such protein fucosylation occurs in the Golgi apparatus and endoplasmic reticulum (specifically O-fucosylation) and is catalyzed by fucosyltransferases. These enzymes utilize GDP-fucose as a donor of the L-fucosyl moiety, which is then transferred onto target proteins (Figure 2).

L-Fucose is perhaps most widely recognized for its presence in the glycans of ABO blood group antigens (see [24] for a review). It is also an essential component of carbohydrate ligands presented by endothelial cells lining the venules, where it facilitates leukocyte rolling on the endothelium. Furthermore, fucosylated antigens are exploited by cancer cells to enhance migration and metastasis (see [29] for a review).

While the importance of GDP-L-fucose biosynthesis in various biological processes is well established [23], the enzymology and biological role of L-fucose degradation remain largely enigmatic. In this review, we summarize current knowledge regarding the enzymological and physiological aspects of L-fucose catabolism in mammals.

## 2. L-Fucose Catabolism in Bacteria

Certain bacterial species, such as *Lactobacillus rhamnosus* and *Escherichia coli*, can utilize L-fucose as a primary carbon and energy source. This capability is mediated by a dedicated L-fucose-inducible operon [30], a system that, among other functions, enables pathogenic bacteria to colonize the mammalian intestine [31].

**Figure 3:**
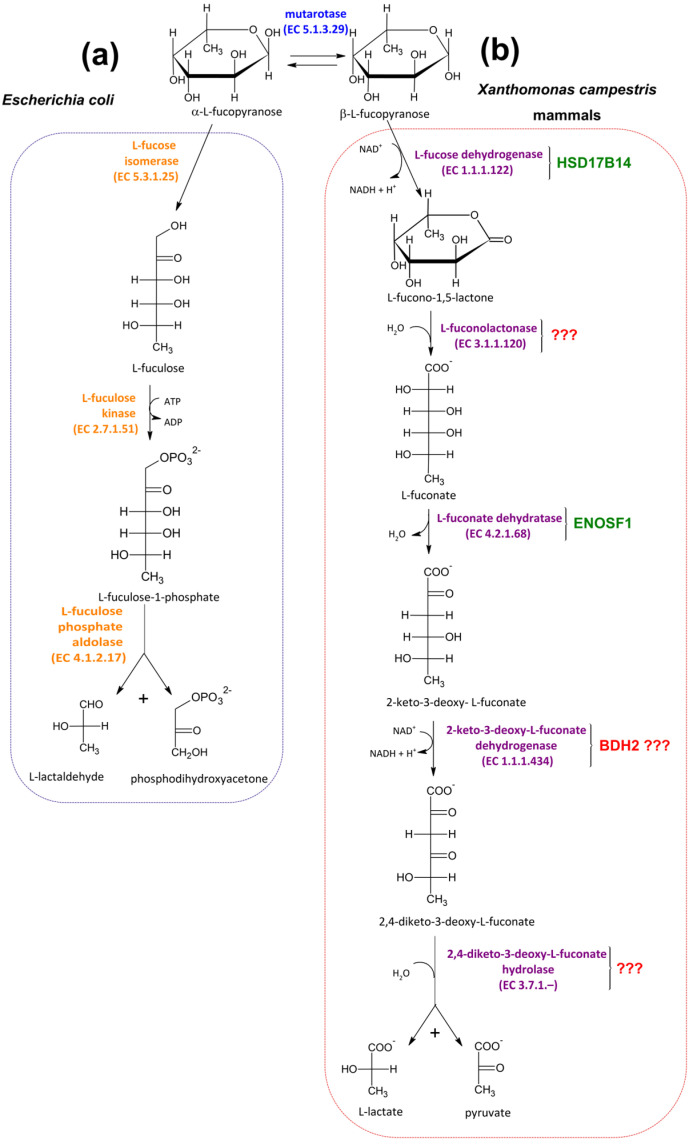
L-Fucose degradation pathways. (**a**) L-fucose can be catabolized by bacteria via either the phosphorylative or (**b**) the non-phosphorylative pathway. The non-phosphorylative pathway is also likely present in certain mammalian species. Bacterial enzymes involved in the phosphorylative and non-phosphorylative routes are highlighted in orange and purple, respectively. Known mammalian enzymes are shown in green, whereas those whose molecular identities remain unknown are indicated by red question marks. BDH2 is the most likely candidate for 2-keto-3-deoxy-L-fuconate dehydrogenase, although this identification requires further experimental validation. Adapted in part with permission from [33]. Copyright 2012 American Chemical Society.

Bacterial L-fucose catabolism varies significantly across species. To date, two distinct L-fucose degradation pathways have been characterized: the phosphorylative (isomerization) pathway, which occurs predominantly in *E. coli*, and the non-phosphorylative (oxidation) pathway, found in *Xanthomonas campestris* [32,33] (Figure 3). Notably, L-fucose does not merely serve as a substrate for energy metabolism; it also functions as a critical signaling molecule in host-microbe interactions [30].

### 2.1. Phosphorylative Pathway

Research has demonstrated that *E. coli* can utilize L-fucose as its sole source of carbon and energy [34]. L-fucose is transported into the cytoplasm via the FucP transporter, a process mediated by proton symport. While FucP is not exclusive to L-fucose, as it can also transport L-galactose and D-arabinose, it does so with significantly lower efficiency [35].

The initial step of the L-fucose phosphorylation pathway in *E. coli* is catalyzed by the mutarotase (FucU, EC 5.1.3.29), which shares 44% sequence identity with its human homolog (FUOM). This enzyme catalyzes the interconversion between the α- and β-anomers of L-fucose, which differ in the orientation of the hydroxyl group at the C1 position [36]. The subsequent step involves the isomerization of α-L-fucopyranose into L-fuculose by L-fucose isomerase (FucI, EC 5.3.1.25). Evidence suggests that the α-anomer is the preferred substrate for this isomerase [37]. The resulting L-fuculose is then phosphorylated to L-fuculose-1-phosphate in a reaction mediated by L-fuculokinase (FucK, EC 2.7.1.51). In the final step of this pathway, L-fuculose-1-phosphate aldolase (FucA, EC 4.1.2.17) catalyzes the cleavage of L-fuculose-1-phosphate into dihydroxyacetone phosphate (DHAP) and L-lactaldehyde [34,38,39]. DHAP can enter glycolysis, whereas the metabolic fates of L-lactaldehyde depend on the aerobic status of the environment in which the bacteria live. Under aerobic conditions, L-lactaldehyde is oxidized to L-lactate in the reaction catalyzed by lactaldehyde dehydrogenase (AldA, EC 1.2.1.22). In the absence of oxygen, L-lactaldehyde is reduced to L-1,2-propanediol by lactaldehyde reductase (FucO, EC 1.1.1.77) [40].

### 2.2. Non-Phosphorylative Pathway

The non-phosphorylative pathway of L-fucose degradation has been identified in several bacterial species, including *Xanthomonas campestris* [32], *Campylobacter jejuni* [41], and *Burkholderia multivorans* [33]. While several enzymes of this pathway have been purified and characterized preliminarily in mammals [32,42,43,44,45,46], the complete molecular framework is most clearly defined in prokaryotes. Furthermore, gene clusters containing homologs of the *X. campestris* fucose degradation genes have been identified in other bacterial species, supporting the widespread occurrence of this pathway in the prokaryotic domain [47].

The pathway is initiated by the oxidation of the β-anomer of L-fucose to L-fucono-1,5-lactone, a reaction catalyzed by an NAD^+^-dependent L-fucose dehydrogenase (EC 1.1.1.122) [32,46,48] (see Figure 3). Interestingly, an alternative bacterial enzyme, D-arabinose (L-fucose) dehydrogenase (EC 1.1.1.116), has been described in *Pseudomonas* sp.; this enzyme catalyzes the oxidation of L-fucose in the presence of NADP^+^ [49], suggesting variability in cofactor preference among bacterial homologs.

Following its formation, L-fucono-1,5-lactone may undergo spontaneous conversion to L-fucono-1,4-lactone [33]. The subsequent step involves the hydrolysis of the lactone to yield L-fuconate. An L-fucono-1,5-lactonase (EC 3.1.1.120) has been identified in *B. multivorans* that displays activity toward both L-fucono-1,5- and L-fucono-1,4-lactone [33]. This enzyme shares 36% protein sequence identity with XCC4066, a putative lactonase from *X. campestris* [33].

In the following step, L-fuconate is dehydrated to 2-keto-3-deoxy-L-fuconate by L-fuconate dehydratase (EC 4.2.1.68) [32,50]. This enzyme was identified at the molecular level and characterized biochemically in *X. campestris* [32]. The resulting 2-keto-3-deoxy-L-fuconate is further metabolized by 2-keto-3-deoxy-L-fuconate dehydrogenase (EC 1.1.1.434). As with other enzymes in this pathway, its activity was initially inferred from studies of L-fucose metabolism in mammalian systems [43,44,51,52], but detailed biochemical characterization has only been performed for the recombinant enzyme from *X. campestris* (XCC4067) [32].

In the final step of the pathway, 2,4-diketo-3-deoxy-L-fuconate—the product of the dehydrogenase reaction—is cleaved into pyruvate and L-lactate by 2,4-diketo-3-deoxy-L-fuconate hydrolase (EC 3.7.1.26) [32].

## 3. L-Fucose Catabolism in Mammals

Early human studies demonstrated that approximately 40% of intravenously injected, labeled L-fucose is readily oxidized to ^14^CO_2_ [53], with a relatively short serum half-life of 100 min [54]. Similar results were observed in cats, guinea pigs, and rabbits, whereas rats appeared unable to effectively catabolize the monosaccharide [55]. These in vivo observations are corroborated by in vitro findings showing that L-fucose is efficiently degraded into pyruvate and L-lactate in pig liver and kidney homogenates; notably, this mammalian metabolic pathway resembles the non-phosphorylative pathway present in certain bacterial species [52].

In mammals, L-fucose is sequestered from the extracellular milieu by a specific membrane transporter, which was recently and unexpectedly identified as glucose transporter 1 (GLUT1) [25]. Furthermore, macropinocytosis contributes to this uptake, distinguishing it from other endocytic pathways [25]. The chemical equilibrium between the α and β anomers of L-fucopyranose (30:70) is rapidly established by the activity of a specific L-fucose mutarotase (FUOM) [56]. Monosaccharide degradation is initiated by L-fucose dehydrogenase, which catalyzes the oxidation of β-L-fucopyranose to L-fucono-1,5-lactone. While this lactone was initially reported to undergo spontaneous hydrolysis to L-fuconate [46,57], recent evidence suggests that this process is likely facilitated by a specific lactonase [48]. Subsequently, L-fuconate dehydratase converts L-fuconate to 2-keto-3-deoxy-L-fuconate [58], which is then oxidized to 2,4-diketo-3-deoxy-L-fuconate by an NAD^+^-dependent dehydrogenase [44]. Finally, 2,4-diketo-3-deoxy-L-fuconate hydrolase completes the pathway, yielding pyruvate and L-lactate, both of which can be further oxidized to CO_2_ through central energy metabolism [52]. Despite the characterization of these steps, the biological relevance of this pathway remains largely unexplored.

### 3.1. L-Fucose Dehydrogenase

L-Fucose dehydrogenase initiates the degradation of β-L-fucopyranose by catalyzing the NAD^+^-dependent oxidation of the sugar to L-fucono-1,5-lactone [57]. This enzyme was partially purified from pig liver and characterized biochemically for the first time by Schachter et al. [46]. In subsequent years, the enzyme was also studied following its isolation from rabbit [42] and sheep liver [59]. These early studies revealed that while the dehydrogenase accepted D-arabinose and L-galactose as substrates, it exhibited a clear preference for L-fucose. Despite these biochemical insights, L-fucose dehydrogenase remained an orphan enzyme until our recent identification of the hydroxysteroid 17-β-dehydrogenase 14 (HSD17B14) protein as the specific mammalian L-fucose dehydrogenase [48].

Human HSD17B14 consists of 270 amino acids and contains a characteristic NAD(H)-binding Rossmann-fold domain [60]. It belongs to the short-chain dehydrogenase/reductase (SDR) superfamily, a group of enzymes that metabolizes a diverse range of compounds in mammals, including steroid hormones, lipids, and xenobiotics [61]. The monomeric molecular mass is approximately 28 kDa; upon cofactor binding, the enzyme assumes its holo form as a homotetramer [62]. Transcriptomic studies indicate that *HSD17B14* mRNA is primarily expressed in the choroid plexus, liver, kidneys, and ovaries, with the protein localizing exclusively to the cytoplasm (Human Protein Atlas; https://www.proteinatlas.org, accessed on 11 May 2026).

HSD17B14 was originally identified as a novel 17-β-hydroxysteroid dehydrogenase that catalyzes the oxidation of the C17-hydroxyl group of estradiol and testosterone to form estrone and androstenedione, respectively [60]. However, the poor kinetic parameters observed in the presence of these steroid substrates suggested they were not the physiological targets of the enzyme. Furthermore, experimental testing of approximately 50 different compounds—including various androgens, estrogens, bile acids, and coenzyme A derivatives—failed to confirm significant enzymatic activity against these metabolites [60]. Notably, recombinant HSD17B14 was unexpectedly found to accept glycerol (used as a cryoprotectant) as a substrate [62]. This finding implied that polyhydroxylated metabolites, such as monosaccharides, might represent the enzyme’s true physiological substrates, though this observation was not initially pursued.

Recent studies in our laboratory [48] led to the definitive molecular identification of HSD17B14 as the mammalian L-fucose dehydrogenase. This was achieved through the extensive purification of the rabbit enzyme using various chromatographic methods followed by tandem mass spectrometric analysis. Throughout the purification process, HSD17B14 emerged as the only viable candidate for the enzyme. This identification was subsequently confirmed by producing recombinant, homogenous rabbit and human HSD17B14 and assessing their activity toward L-fucose and structurally related compounds. The recombinant enzymes preferentially oxidized L-fucose, followed by D-arabinose and L-galactose, whereas D-threose and the 2-, 3-, and 4-epimers of D-arabinose were poor substrates. This substrate specificity indicates that HSD17B14 acts primarily on the pyranose form of the sugar and that the configuration of the hydroxyl groups at C-2 through C-4 is critical for enzymatic activity.

Additionally, β-estradiol proved to be an exceptionally poor substrate. Recombinant rabbit and human enzymes were approximately 1000- to 2300-times more effective toward 5 µM L-fucose than 5 µM β-estradiol, effectively excluding the latter as a physiological substrate for HSD17B14. Importantly, recombinant HSD17B14 was found to oxidize L-fucose to L-fucono-1,5-lactone, which is unstable and rapidly non-enzymatically transforms into L-fucono-1,4-lactone. The latter is a slow-hydrolyzing compound that eventually forms L-fuconate. Because we observed the accumulation of L-fucono-1,4-lactone rather than L-fuconate during the enzymatic reaction—contrary to the original suggestions by Schachter et al. [46]—we concluded that a specific lactonase must be present in mammalian cells to convert L-fucono-1,4-lactone to L-fuconate. L-Fuconate then serves as the substrate for L-fuconate dehydratase.

### 3.2. L-Fuconolactonase

**Figure 4:**
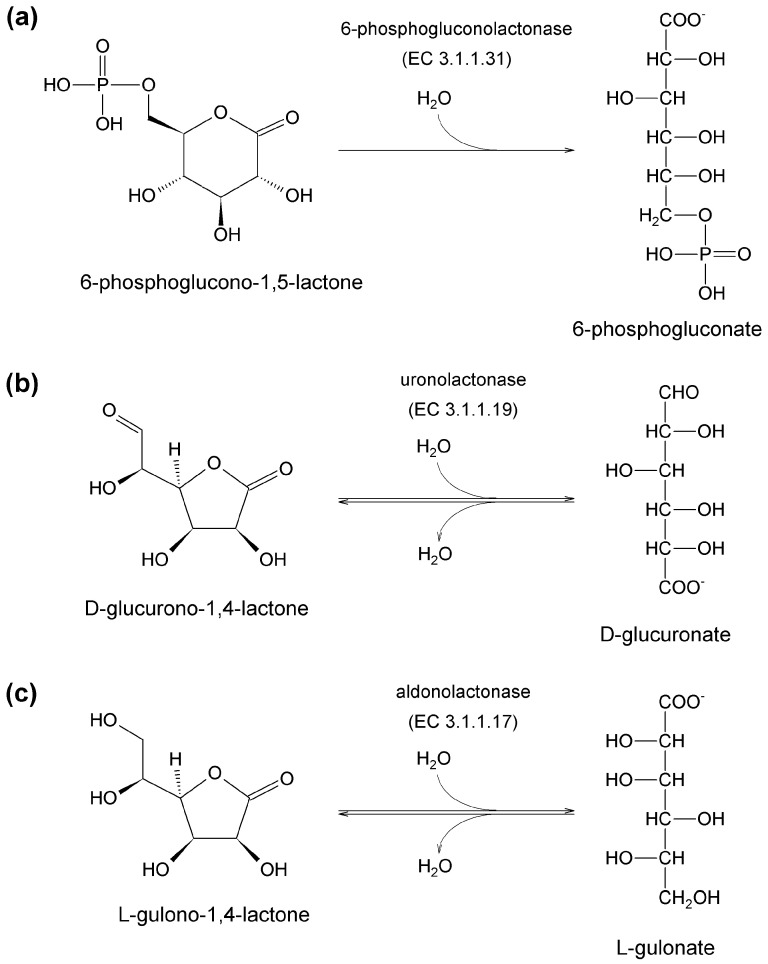
Prototypic reactions catalyzed by mammalian lactonases identified to date. Shown are the reactions performed by (**a**) 6-phosphogluconolactonase, (**b**) uronolactonase, and (**c**) aldonolactonase. Uronolactonase is a microsomal enzyme, whereas 6-phosphogluconolactonase and aldonolactonase are cytosolic.

L-Fuconolactonase has never been detected in mammalian tissues. To date, three lactonases are known to be active against sugar acid lactones in mammals: 6-phosphogluconolactonase, uronolactonase, and aldonolactonase (Figure 4).

6-Phosphogluconolactonase (6-PGL; EC 3.1.1.31) is the second enzyme of the oxidative branch of the pentose phosphate pathway (PPP) (see Figure 4). It is located in the cytoplasm and is prevalent in various tissues—particularly those involved in fatty acid synthesis—and in erythrocytes [63], where the PPP serves as a primary source of reducing power (NADPH). The pathway also provides essential precursors for the synthesis of nucleotides and amino acids [64].

The product of glucose-6-phosphate dehydrogenase (G6PDH) activity—6-phosphoglucono-1,5-lactone (6-phosphoglucono-δ-lactone) can undergo spontaneous hydrolysis into 6-phosphogluconate or rapidly rearrange into 6-phosphoglucono-1,4-lactone (6-phosphoglucono-γ-lactone). The latter is a more stable form that either resists spontaneous hydrolysis or hydrolyzes significantly slower than the 1,5-lactone. 6-PGL acts specifically on the 1,5-lactone and is thought to prevent the accumulation of the stable 1,4-form [65]. Furthermore, the enzyme may prevent potentially deleterious reactions between electrophilic 6-phosphoglucono-1,5-lactone and intracellular nucleophiles [65,66]. 6-PGL appears highly specific for 6-phosphogluconolactone [63]. Additionally, 6-PGL activity is independent of metal ions [67].

There are no disorders known to be caused by 6-PGL deficiency except for a case described by Beutler et al. [68], in which a partial 6-PGL deficiency combined with partial deficiency of G6PDH was suggested to be the underlying cause of hemolytic anemia present in a patient. Conversely, increased levels of 6-PGL have been reported in various cancers where it is associated with reduced oxidative stress and accelerated cell proliferation [69,70].

Uronolactonase (EC 3.1.1.19) has not been extensively studied. It is localized in the microsomal fraction of the mammalian liver and is also present in small quantities in the adrenal glands of guinea pigs. Uronolactonase requires a divalent metal ion for its catalytic activity. To date, D-glucurono-1,4-lactone (D-glucurono-γ-lactone) is the only known sugar acid lactone hydrolyzed by this enzyme, yielding D-glucuronate as the reaction product (see Figure 4) [71,72].

**Figure 5:**
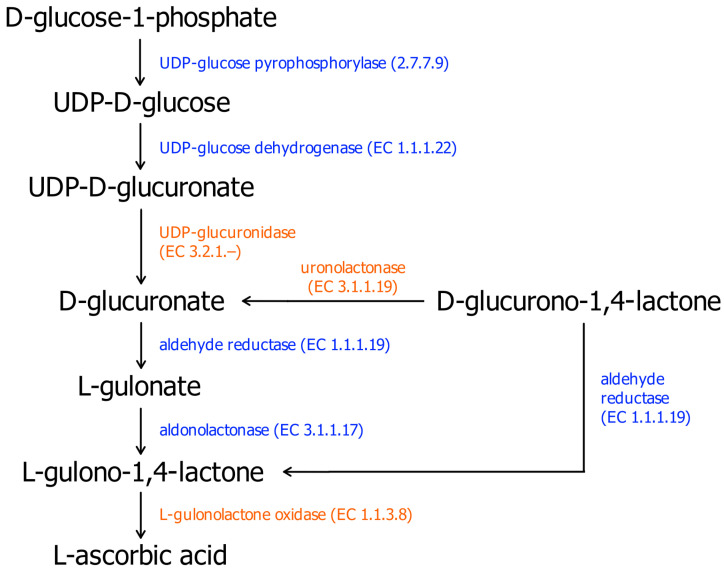
Biosynthesis of vitamin C in mammals. Enzymes located in the cytoplasm are colored blue; microsomal enzymes are colored orange.

The enzyme can facilitate the synthesis of L-ascorbic acid from D-glucurono-1,4-lactone by producing D-glucuronate, which is subsequently converted to L-gulonate by aldehyde reductase. L-gulonate then undergoes lactonization by aldonolactonase to form L-gulono-1,4-lactone (L-gulono-γ-lactone), the substrate for the terminal enzyme of L-ascorbic acid biosynthesis: L-gulonolactone oxidase (Figure 5) [73,74].

While aldehyde reductase can reduce glucuronolactone directly into gulonolactone [75], the in vivo metabolic flux through this alternative pathway is relatively small [76]. It is generally accepted that the pathway utilizing D-glucuronate and L-gulonate as intermediates is more prevalent [73]. Currently, uronolactonase has not yet been molecularly identified, consequently, the full physiological role of this enzyme remains uncertain.

Aldonolactonase (EC 3.1.1.17), also known as gluconolactonase, gulonolactonase, regucalcin, or senescence marker protein 30 (SMP30), is a lactonase characterized by broad substrate specificity (see Figure 4). It is highly conserved throughout vertebrate evolution [77] and is localized within both the cytoplasm and the nuclei of cells [78]. While the protein is primarily expressed in the mammalian liver and kidneys, it is also present to a lesser extent in various other tissues [72,79]. Aldonolactonase hydrolyzes a wide array of D- and L-aldonic acid lactones as well as organophosphates, its activity is dependent on divalent metal ions [72,80,81].

Aldonolactonase is a multifunctional protein. In the L-ascorbic acid biosynthetic pathway of vertebrates, it catalyzes the lactonization of L-gulonic acid [74,80]. Additionally, aldonolactonase could play a potential protective role against protein glycation by aldonic acid lactones, as Lindsay et al. [82] demonstrated that D-glucono-1,5-lactone (D-glucono-δ-lactone) causes hemoglobin glycation in vitro and in vivo more potently than D-glucose.

Furthermore, aldonolactonase exhibits functions that extend far beyond its enzymatic activity. These include the regulation of intracellular Ca^2+^ levels, anti-apoptotic and anti-proliferative effects, protection against oxidative stress, and others (reviewed in [79,83,84,85]). Reduced expression of aldonolactonase has been reported in multiple types of human cancers; the protein appears to function as a tumor suppressor, with low levels promoting carcinogenesis (reviewed in [86]). Decreased aldonolactonase levels have also been observed in the livers of patients with nonalcoholic fatty liver disease (NAFLD) [87] and in the kidney tissue of patients with diabetic nephropathy [88]. While no human genetic disorders have been linked to mutations in the aldonolactonase gene, the effects of aldonolactonase knockout have been extensively investigated in murine models (reviewed in [79,81,85]).

L-fuconolactonase appears to be essential for efficient L-fucose degradation ([48] see Section 3.1). Although this activity has been confirmed in the L-fucose catabolic pathway of *B. multivorans* ([33]; see Section 2), neither the specific activity nor the enzyme responsible has been characterized in mammals to date. While the molecular identity of this lactonase remains unknown, our preliminary studies confirm the presence of such hydrolase activity in rabbit liver. It might be performed by an uncharacterized “orphan” enzyme, potentially a misassigned hydrolase. Alternatively, one of the known lactonases discussed above may exhibit secondary fuconolactonase activity. Thus, the most direct approach to identifying L-fuconolactonase is to express recombinant forms of each known mammalian lactonase in *E. coli* or HEK293T cells and evaluate their activity toward L-fucono-1,4-lactone using a spectrophotometric assay [33]. Should this approach fail, a more classical strategy could be employed to purify the enzyme from porcine liver [52], identify all hydrolases in the purified preparation via mass spectrometry, and select the most promising candidates. Subsequently, the recombinant form of the selected candidate can be produced and its activity toward L-fucono-1,4-lactone verified.

### 3.3. L-Fuconate Dehydratase

L-Fuconate dehydratase (EC 4.2.1.68) catalyzes the dehydration of L-fuconate to 2-keto-3-deoxy-L-fuconate within the non-phosphorylative pathway of L-fucose degradation [32,50]. Early studies in mammals allowed for the partial purification and preliminary characterization of this enzymatic activity, demonstrating its presence as part of a putative catabolic pathway for L-fucose in pork liver [52,58]. The native enzyme, purified through a multistep procedure, exhibited a pH optimum of approximately 7.0 and required Mg^2+^ for catalytic activity. Substrate specificity analyses indicated that L-fuconate is the preferred substrate, although activity toward structurally related sugar acids, such as D-fuconate and D-arabonate, was also observed. The reaction product was identified as 2-keto-3-deoxy-L-fuconate via spectrophotometric assays and further confirmed by NMR analysis [58].

While the mammalian enzyme has long been considered an orphan enzyme, the bacterial L-fuconate dehydratase was subsequently identified as a product of the *fucD* gene and biochemically characterized in *X. campestris* (XCC4069) [32], providing detailed insights into its catalytic properties. Using the recombinant enzyme, ^1^NMR studies confirmed that L-fuconate is dehydrated to form 2-keto-3-deoxy-L-fuconate. While L-fuconate was identified as the most relevant substrate, residual activity was also detected for five structurally related sugar acids: L-galactonate, D-arabinonate, L-talonate, D-ribonate, and D-altronate. Furthermore, the molecular structure of the FucD enzyme was resolved, providing critical mechanistic insights into its catalytic cycle and substrate specificity [32]. Although the homologous reverse thymidylate synthase protein (rTSβ) from *Homo sapiens* was not isolated or assayed in that specific study, its 52% sequence identity with the protein encoded by *XCC4069* and the conservation of key substrate-binding residues led to the hypothesis that rTSβ also catalyzes the dehydration of L-fuconate [32].

In contrast to this earlier hypothesis [32], further comparative sequence analysis of bacterial homologs and functional studies identified reverse thymidylate synthase rTSγ, rather than rTSβ, as the human L-fuconate dehydratase. rTSγ, a 50 kDa protein, corresponds to Enolase Superfamily Member 1 (ENOSF1) and is one of three splice variants identified in *H. sapiens*, alongside rTSα and rTSβ. The gene encoding the rTSγ protein was originally identified as a source of antisense RNAs for the adjacent thymidylate synthase (*TYMS*) gene [89], which potentially promote the degradation of *TYMS* mRNA [90]. While it was initially postulated to be a mitochondrial protein [91], subsequent research has questioned this localization [50]. Transcriptomic data indicate that human *ENOSF1* mRNA is primarily expressed in the thyroid, kidney, choroid plexus, liver, adrenal gland, and pituitary gland (Human Protein Atlas; https://www.proteinatlas.org, accessed on 11 May 2026).

While the physiological roles of these isoforms remain largely uncharacterized, biochemical analysis of the recombinant rTSγ protein confirmed its dehydratase activity toward L-fuconate. Kinetic analyses revealed that rTSγ exhibits activity toward several structurally related sugar acids, including L-galactonate, L-arabinarate, and D-arabinonate. However, the highest catalytic efficiency was observed for L-fuconate, suggesting that this sugar acid serves as the physiological substrate [50]. These findings, combined with sequence conservation (52% identity, 71% similarity), structural features typical of the enolase superfamily, and kinetic properties, established ENOSF1 as the human counterpart of the bacterial L-fuconate dehydratases initially characterized in *X. campestris* [32,50] and later in *Paraburkholderia mimosarum* [92]. Although ENOSF1 has been molecularly identified as an L-fuconate dehydratase, its precise physiological role in human L-fucose metabolism has yet to be fully elucidated.

### 3.4. 2-Keto-3-deoxy-L-fuconate Dehydrogenase

2-Keto-3-deoxy-L-fuconate dehydrogenase (EC 1.1.1.434) catalyzes the NAD^+^-dependent oxidation of 2-keto-3-deoxy-L-fuconate in a subsequent step of the non-phosphorylative pathway of L-fucose degradation [32,52]. Early mammalian studies identified this enzymatic activity in the soluble fraction of pork liver, supporting the existence of a catabolic route for L-fucose [52]. The enzyme was purified to near-homogeneity through a multistep procedure including affinity chromatography on NAD^+^-agarose, achieving an approximately 3000-fold purification while retaining catalytic activity [44]. Subsequent biochemical characterization, including kinetic and substrate specificity studies, revealed that the enzyme is NAD^+^-dependent and exhibits the highest activity toward 2-keto-3-deoxy-L-fuconate. However, it also accepts structurally related sugar acid derivatives, such as 2-keto-3-deoxy-D-arabonate, 2-keto-3-deoxy-D-gluconate, and 2-keto-3-deoxy-D-galactonate, indicating a relatively broad substrate specificity [51].

Similarly to L-fuconate dehydratase, this enzyme was molecularly identified and biochemically characterized in *X. campestris*, where the corresponding gene (*XCC4067*) was assigned based on its genomic context [32]. Functional characterization of the recombinant XCC4067 enzyme confirmed its NAD^+^-dependent dehydrogenase activity toward 2-keto-3-deoxy-L-fuconate, consistent with its role in the non-phosphorylative pathway. Paralleling the earlier mammalian studies, the bacterial enzyme also demonstrated activity toward structurally related 2-keto-3-deoxy sugar acids, such as 2-keto-3-deoxy-L-galactonate and 2-keto-3-deoxy-D-arabinonate. Nevertheless, kinetic properties indicated that 2-keto-3-deoxy-L-fuconate is likely the physiological substrate [32]. These findings, alongside the mammalian data, provide evidence for the enzymatic conversion of 2-keto-3-deoxy-L-fuconate across both bacteria and mammals [32,44,51,52]. However, the molecular identity of the mammalian 2-keto-3-deoxy-L-fuconate dehydrogenase remained unknown, leaving it classified as an “orphan enzyme”. Intriguingly, results from recent studies suggest that mammalian 4-oxo-L-proline reductase (BDH2) may indeed be the sought-after dehydrogenase.

The molecular identity of mammalian 4-oxo-L-proline reductase (EC 1.1.1.104) was elucidated through a multistep purification process combined with proteomic analysis. Following the purification of the native enzyme from rat kidney, tandem mass spectrometry identified type 2 (R)-β-hydroxybutyrate dehydrogenase (BDH2) as the only feasible candidate co-eluting with the enzymatic activity [93]. To validate this identification, both rat and human recombinant BDH2 were produced, purified to homogeneity, and tested in specific enzymatic assays. Both BDH2 homologs exhibited NADH-dependent reduction of 4-oxo-L-proline to *cis*-4-hydroxy-L-proline, which was subsequently confirmed by mass spectrometry analysis of the reaction products [93].

BDH2 is a 245-amino acid, 27 kDa cytosolic protein that assembles into a homotetramer in its native form [94]. Transcriptomic data indicate that human *BDH2* mRNA is primarily expressed in the kidneys, with levels approximately five-fold lower in the midbrain, choroid plexus, liver, and duodenum; however, transcripts are also detectable across various other tissues (Human Protein Atlas; https://www.proteinatlas.org, accessed on 11 May 2026).

Biochemical characterization of the recombinant enzyme revealed a strong preference for cyclic substrates, with high catalytic efficiency toward 4-oxo-L-proline. Notably, only minimal activity was detected with (R)-β-hydroxybutyrate—the previously postulated endogenous substrate—implying that it is unlikely to be the physiologically relevant substrate for this enzyme [93]. Cellular studies demonstrated that wild-type HEK293T cells could produce *cis*-4-hydroxy-L-proline from 4-oxo-L-proline, whereas *BDH2*-deficient HEK293T cells were unable to metabolize it. Additionally, BDH2 was shown to catalyze the reversible oxidation of *cis*-4-hydroxy-L-proline to 4-oxo-L-proline [93].

**Figure 6:**
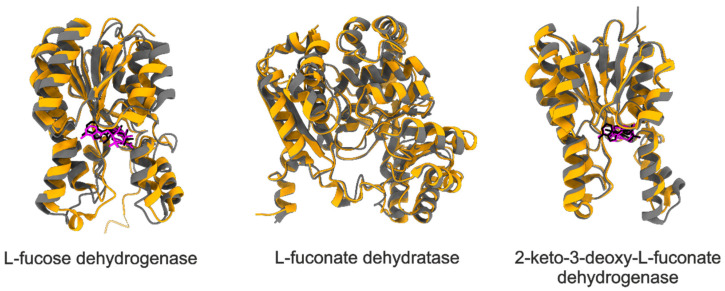
Comparison of the three-dimensional structures of human L-fucose dehydrogenase, L-fuconate dehydratase, and the putative 2-keto-3-deoxy-L-fuconate dehydrogenase with those of their bacterial homologues. The superpositions of ribbon representations of the human (orange) and bacterial (gray) enzymes clearly indicate highly similar fold architectures. Human L-fucose dehydrogenase (HSD17B14, PDB: 5HS6), L-fuconate dehydratase (ENOSF1, PDB: 4A35), and the putative 2-keto-3-deoxy-L-fuconate dehydrogenase (BDH2, PDB: 2AG5) were aligned to their bacterial homologues: *B. multivorans* L-fucose dehydrogenase (LFUCD_BURM1, PDB: 4GVX), *X. campestris* L-fuconate dehydratase (XCC4069, PDB: 2HXU), and 2-keto-3-deoxy-L-fuconate dehydrogenase from *Herbaspirillum huttiense* (PDB: 8Y11). Human dehydrogenases are shown in complex with NAD (black sticks), whereas the bacterial dehydrogenases are illustrated with bound NAD(P) (magenta sticks). All models were prepared using UCSF ChimeraX version: 1.11.1 [95].

Comparative sequence analysis of the human BDH2 protein against bacterial databases revealed significant similarity to bacterial 2-keto-3-deoxy-L-fuconate dehydrogenases. A detailed comparison of the amino acid sequences of human BDH2 and the XCC4067 enzyme from *X. campestris* demonstrated a high degree of similarity (68%) and identity (55%), suggesting that BDH2 may function as the 2-keto-3-deoxy-L-fuconate dehydrogenase in the mammalian L-fucose degradation pathway. This finding was further strengthened by a striking structural similarity between the human BDH2 enzyme and bacterial 2-keto-3-deoxy-L-fuconate dehydrogenase, mirroring the conservation observed between other human and bacterial enzymes of this pathway (Figure 6).

**Figure 7:**
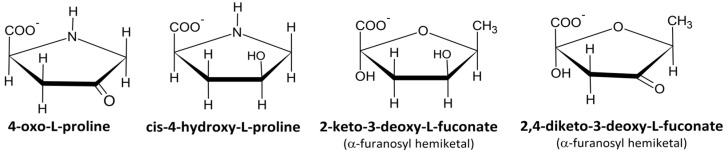
Structural comparison of 4-oxo-L-proline, *cis*-4-hydroxy-L-proline, and hemiketal forms of 2-keto-3-deoxy-L-fuconate and 2,4-diketo-3-deoxy-L-fuconate.

Structural comparisons of 4-oxo-L-proline, *cis*-4-hydroxy-L-proline, and the hemiketal form of 2-keto-3-deoxy-L-fuconate revealed notable similarities (Figure 7). Both cyclic compounds resemble the hemiketal intermediates, suggesting this structural feature is critical for substrate recognition. Importantly, 2-keto-3-deoxy-L-fuconate and related sugar acids can form intramolecular hemiketal structures, potentially serving as alternative substrates for BDH2, much like the bacterial counterpart [32]. Recent structural and comparative studies further support this link [47]. Like bacterial 2-keto-3-deoxy-L-fuconate dehydrogenases, BDH2 belongs to the SDR superfamily and shares conserved sequence features. Structural modeling indicates that both the bacterial enzymes and BDH2 can accommodate substrates in cyclic conformations, with evidence of overlapping substrate tolerance [47]. Consequently, BDH2 emerges as a strong candidate for the orphan 2-keto-3-deoxy-L-fuconate dehydrogenase. However, current evidence indicates that BDH2 exhibits broad substrate promiscuity, catalyzing the metabolism of a wide spectrum of metabolites, including 3-hydroxybutyrate [94], 4-oxo-L-proline [93], 2,5-dihydroxybenzoate (2,5-DHBA) [96], and 2-keto-3-deoxy-L-fuconate [47]. This points to its involvement in a diverse range of metabolic processes, such as ketone body metabolism [94], the detoxification of 4-oxo-L-proline [93], the synthesis of the mammalian siderophore (2,5-DHBA) [96], and L-fucose catabolism [47]. Which of these functions is physiologically paramount remains an open question. Ongoing studies focus on the biochemical characterization of BDH2 to address this issue, though definitively resolving its precise physiological contribution to human metabolism will ultimately require metabolic studies utilizing *BDH2*-knockout cell lines or animal models.

### 3.5. 2,4-Diketo-3-deoxy-L-fuconate Hydrolase

2,4-Diketo-3-deoxy-L-fuconate hydrolase (EC 3.7.1.-) is thought to catalyze the terminal step of the non-phosphorylative L-fucose degradation pathway in the mammalian liver and kidneys. This reaction converts 2,4-diketo-3-deoxy-L-fuconate into pyruvate and L-lactate. The existence of this enzymatic activity was first proposed by Schachter and colleagues, who reconstructed the pathway in mammalian liver extracts through a series of biochemical analyses; however, they were unable to isolate the specific hydrolase responsible for this final catabolic step [44,51,68]. Crucially, the authors noted that 2,4-diketo-3-deoxy-L-fuconate is a highly unstable metabolite, a factor that has significantly hampered more in-depth studies of the enzyme.

Nevertheless, this missing step was further investigated in a subsequent study by Chan et al., who demonstrated that L-fucose can be metabolized to L-lactate in mammalian liver preparations [52]. By using radioisotopically labeled intermediates, the authors showed that carbon atoms derived from 2-keto-3-deoxy-L-fuconate are quantitatively recovered in L-lactate, providing strong evidence for a complete metabolic pathway. Based on these findings, they proposed that the diketo intermediate undergoes cleavage—most likely between the C3 and C4 positions—yielding smaller metabolites that are subsequently converted to L-lactate. However, despite clear functional evidence for this reaction, the enzyme responsible for catalyzing the cleavage of 2,4-diketo-3-deoxy-L-fuconate was neither isolated nor biochemically characterized.

Conversely, studies on bacterial catabolic pathways for pentose and deoxyhexose sugars have postulated that 2,4-diketo-3-deoxy-L-fuconate is broken down into L-lactate and pyruvate via a reaction catalyzed by a specific hydrolase [40,97,98]. The enzyme responsible for this activity in *H. huttiense* was identified as the C785_RS20550 protein [97], while the *Sphingomonas* sp. LRA6 protein was found to be a 2,4-diketo-3-deoxy-L-rhamnonate hydrolase, which was subsequently crystallized and biochemically characterized in detail [99]. Notably, both bacterial proteins belong to the fumarylacetoacetate hydrolase (FAH) family.

**Figure 8:**
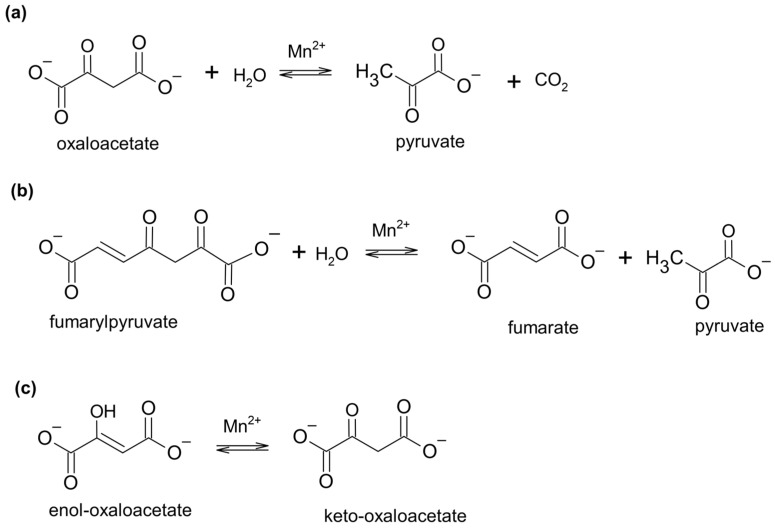
Diverse enzymatic activities of the human FAHD1 protein. FAHD1 has been shown to catalyze: (**a**) the decarboxylation of oxaloacetate; (**b**) the hydrolysis of acylpyruvates, such as fumarylpyruvate and acetylpyruvate; and (**c**) the tautomerization of enol-oxaloacetate to its physiological keto form.

Based on amino acid sequence similarity between these bacterial proteins and human orthologs, it was hypothesized that the human fumarylacetoacetate hydrolase domain-containing protein 1 (FAHD1) might account for the 2,4-diketo-3-deoxy-L-fuconate hydrolase activity [47,99]. Mammalian FAHD1 was initially identified as an oxaloacetate decarboxylase (EC 4.1.1.112), catalyzing the decarboxylation of oxaloacetate to pyruvate and CO_2_ [100], and as an acylpyruvate hydrolase (EC 3.7.1.5) capable of cleaving both fumarylpyruvate and acetylpyruvate to yield pyruvate and either fumarate or acetate, respectively (Figure 8) [101]. More recently, FAHD1 and the closely related proteins FAHD2A and FAHD2B were shown to function as tautomerases that convert enol-oxaloacetate—a potent inhibitor of succinate dehydrogenase—to the physiological keto form of oxaloacetate within the mitochondria [101,102]. Consequently, FAHD1 does not appear to be a plausible 2,4-diketo-3-deoxy-L-fuconate hydrolase, as L-fucose degradation is thought to occur in the cytosolic compartment, whereas FAHD1 is localized to the mitochondria. Furthermore, there is currently no direct experimental evidence demonstrating that FAHD1 exhibits activity toward 2,4-diketo-3-deoxy-L-fuconate. Thus, the molecular identity of the mammalian 2,4-diketo-3-deoxy-L-fuconate hydrolase remains unknown. Resolving the identity of this enzyme is essential for completing the description of the L-fucose catabolic pathway. The most direct approach to achieve this goal is the sequential purification of the enzyme from a suitable source, such as porcine tissue [52], followed by the identification of the proteins present in the purified preparation. From the resulting protein list, the most promising hydrolase candidates can be selected. Subsequently, a recombinant form of the candidate can be expressed in *E. coli* or HEK293T cells, purified, and its activity toward 2,4-diketo-3-deoxy-L-fuconate verified using a simple spectrophotometric assay coupled with L-lactate dehydrogenase. In our laboratory, this approach has proven highly successful for identifying various orphan enzymes over more than a decade of research [11,18,58,93]. However, a potential challenge lies in obtaining the necessary quantities of 2,4-diketo-3-deoxy-L-fuconate to serve as the substrate for activity assays during the purification process.

## 4. Outlook

Over five decades have elapsed since Schachter and colleagues demonstrated that L-fucose is catabolized into L-lactate and pyruvate through a specific metabolic pathway present in the liver and kidneys of several mammals [52]. Although most enzymes contributing to this pathway have been isolated and biochemically characterized to some extent from mammalian tissues, L-fucose dehydrogenase (HSD17B14) and L-fuconate dehydratase (ENOSF1) remain the only molecularly characterized (deorphaned) mammalian enzymes to date. Concurrently, BDH2 represents a highly promising candidate for mammalian 2-keto-3-deoxy-L-fuconate dehydrogenase (Table 1).

Importantly, the apparent absence of direct structural or sequence homologs of bacterial L-fuconolactonases and 2,4-diketo-3-deoxy-L-fuconate hydrolases in mammalian proteomes does not necessarily imply that these enzymatic activities are missing in mammals. Instead, this may indicate that, over evolutionary time, specific bacterial enzymes were replaced by functional mammalian equivalents. These mammalian enzymes likely catalyze analogous reactions despite lacking a direct evolutionary relationship to their bacterial counterparts.

While the designation of HSD17B14 and ENOSF1 as L-fucose dehydrogenase and L-fuconate dehydratase, respectively, was based on rigorous biochemical analyses—including the determination of substrate specificity, catalytic properties, and product verification [48,50]—these findings could be further strengthened by generating knockout mammalian cell lines. Such cell lines would serve as valuable tools for investigating the functional and metabolic consequences of disrupting the L-fucose degradation pathway. For instance, utilizing ^13^C-labeled L-fucose in wild-type and knockout cell cultures would enable researchers to track the flux of pathway intermediates, trace their final metabolic fates, and assess the broader impacts of a metabolic block within the pathway.

However, such metabolic studies may prove highly challenging due to the lack of human cell lines that preserve the complete pathway along with all its constituent enzymes. Indeed, numerous human cell lines are derived from malignancies; these lines are frequently genetically and metabolically dysregulated and fail to maintain intact biochemical pathways. The scarcity of human gluconeogenic cell lines capable of producing and releasing glucose serves as an illustrative example of this limitation [103]. Consequently, the molecular identification of all enzymes contributing to the L-fucose degradation pathway is a critical milestone that must be achieved prior to undertaking more complex metabolic and genetic studies to elucidate the physiological role of this pathway.

The physiological role of this catabolic route remains enigmatic. In human and other mammalian cells, L-fucose is a valuable metabolite primarily utilized for the synthesis of GDP-L-fucose via the salvage pathway (see Figure 2), which is essential for glycoprotein synthesis. However, cells are not solely dependent on an exogenous supply of this monosaccharide, as GDP-L-fucose can also be synthesized from mannose through the de novo pathway. Notably, the metabolic flux of both pathways is regulated by feedback inhibition; the accumulation of GDP-L-fucose strongly inhibits both GDP-mannose 4,6-dehydratase in the de novo pathway [104] and L-fucokinase in the salvage pathway [105]. Thus, it can be hypothesized that an intracellular excess of L-fucose is diverted toward energy production, with L-fucokinase acting as a metabolic switch that directs the monosaccharide either toward GDP-L-fucose formation or oxidation to CO_2_. This notion is supported by the presence of the pathway in energy-demanding tissues such as the liver and kidney. Conversely, this hypothesis is challenged by the fact that L-fucose has limited bioavailability in the standard human diet and its concentration in human serum is low (≈1.7 µM) [25], suggesting it is unlikely to serve as a significant fuel source.

The expression levels of L-fucose dehydrogenase (HSD17B14), L-fuconate dehydratase (ENOSF1), and the putative mammalian 2-keto-3-deoxy-L-fuconate dehydrogenase (BDH2) are notably high in human hepatocytes, renal proximal tubules, and the choroid plexus epithelium (Human Protein Atlas; https://www.proteinatlas.org, accessed on 16 June 2026), suggesting that the L-fucose degradation pathway is primarily localized within these tissues. This expression profile is further supported by histochemical analyses confirming the abundant presence of these enzymes in human hepatocytes and renal proximal tubules, although corresponding protein-level data for the choroid plexus epithelium are currently unavailable. Additionally, this enzyme profile can be detected at varying levels in other tissues, including colon glandular cells and Leydig cells (Human Protein Atlas, accessed on 16 June 2026).

Importantly, hepatocytes, renal proximal tubules, and the choroid plexus epithelium are also characterized by a rapid turnover of fucosylated glycans [106,107,108]. Thus, it is plausible that the L-fucose degradation pathway can serve to prevent the intracellular accumulation of free L-fucose in cells involved in dynamic glycan remodeling. By maintaining a low intracellular concentration of this monosaccharide, the pathway may facilitate its transport out of lysosomes, prevent the feedback inhibition of lysosomal α-L-fucosidases by L-fucose [109,110], and ultimately ensure efficient lysosomal defucosylation (see Figure 2). Indeed, histological examinations have revealed increased vacuolization in the hepatocytes of *HSD17B14* knockout animals compared with wild-type littermates [111]. It is tempting to speculate that these vacuoles represent enlarged, overloaded lysosomes.

Alternatively, the pathway may function to prevent the extracellular accumulation of free L-fucose. For example, efficient absorption of L-fucose from the intestine and its subsequent degradation in the liver may be crucial for maintaining the optimal composition of the intestinal microbiome. In the mammalian gut, L-fucose is a major component of mucin glycoproteins and is highly abundant in the intestinal epithelium [112]. Intestinal commensal microbiota (e.g., *Bacteroides* and *Bifidobacterium*) produce α-L-fucosidases that allow them to harvest L-fucose from the mucosa and utilize this monosaccharide as a source of carbon and energy (for review, [113]). Other intestinal commensal species, such as *Escherichia coli* or *Lactobacillus rhamnosus*, do not secrete extracellular α-L-fucosidases but are nevertheless capable of fermenting the L-fucose released by neighboring microbes [114]. Metabolites generated by microbial L-fucose fermentation—including acetic acid, lactic acid, and 1,2-propanediol—are subsequently converted by other intestinal bacteria into butyric and propionic acids. These short-chain fatty acids are largely absorbed by host epithelial cells, serving as both an energy source and vital signaling molecules [113]. Thus, the human intestine continuously supplies L-fucose via fucosylated glycans to sustain microbial growth and shape the microbiome. However, free L-fucose can also be exploited by opportunistic pathogens. For instance, *Campylobacter jejuni* utilizes free L-fucose to gain a competitive advantage during infection [41]. Furthermore, gastrointestinal pathogens such as enterohemorrhagic *E. coli* (EHEC) possess a specialized free L-fucose-sensing system that regulates virulence gene expression and is required for robust colonization of the mammalian intestine [31]. Consequently, tight control of free L-fucose levels in the intestine appears to be of importance, not only for stabilizing the commensal microbiome but also for defending against pathogen invasion.

By extension, the pathway may facilitate the clearance of L-fucose from body fluids, which is consistent with its presence in the mammalian liver and kidneys [52]. The physiological rationale underlying this clearance may be to limit the progression and severity of bacterial infections. For example, maintaining low serum L-fucose concentrations prevents its excessive excretion into urine, which may limit bacterial growth in the urinary tract and reduce the incidence of infections. Furthermore, more complex pathogenic mechanisms might be involved. For instance, *Streptococcus pneumoniae* is a major human pathogen and the primary etiological agent of severe diseases such as pneumonia, bacteremia, and meningitis [114]. This bacterium produces and secretes two complementary α-L-fucosidases that defucosylate histo-blood group antigens to “uncap” fucosylated host glycans, enabling their complete degradation by secondary enzymes [115]. Intriguingly, although *S. pneumoniae* possesses a complete set of intracellular enzymes that convert L-fucose to dihydroxyacetone, the bacterium is unable to grow on L-fucose and apparently cannot utilize this monosaccharide as a carbon and energy source [116]. Thus, it has been hypothesized that this fucose-processing pathway functions as a sensing mechanism for fucose-containing glycans by harvesting fucose and metabolizing it into intermediates that act as intracellular signaling molecules [116]. More importantly, it cannot be entirely excluded that similar signaling pathways, involving L-fucose catabolites as signaling molecules, operate in human cells. In this scenario, the putative mammalian L-fucose degradation pathway would serve sensing and metabolic regulatory purposes rather than merely functioning in the clearance or consumption of this monosaccharide. In fact, it was shown that the presence of free L-fucose in the cell culture medium stimulates enterocyte-like Caco-2 cells to secrete a panel of cytokines, including TNF-α, IL-5, and IL-12 [117]. Consequently, the accumulation of free fucose may serve as a physiological signal indicating the invasion of mucin-hydrolyzing microbial cells and the subsequent disruption of the mucosal barrier. In light of these observations, it is certainly worth verifying whether these effects are triggered by L-fucose itself or by its breakdown products released into the extracellular milieu.

Finally, the clearance of L-fucose from body fluids may also limit its non-enzymatic adduction to proteins. Similarly to other monosaccharides, L-fucose can cause spontaneous, non-enzymatic glycation of proteins [118], forming ketoamine adducts. These adducts likely adopt a closed-ring furanose structure, as previously demonstrated for glucose-glycated albumin [119]. One may speculate that these non-enzymatically formed fucofuranosyl proteins could interact with receptors specific for L-fucosyl-glycans, thereby disrupting physiological signaling.

## 5. Conclusions

In conclusion, L-fucose dehydrogenase (HSD17B14) and L-fuconate dehydratase (ENOSF1) currently remain the only molecularly characterized enzymes of the putative mammalian L-fucose degradation pathway, whereas BDH2 represents a highly promising candidate for mammalian 2-keto-3-deoxy-L-fuconate dehydrogenase. Comprehensively identifying the remaining constituent enzymes is critical to mapping the specific cells and tissues in which this pathway operates, thereby elucidating its physiological significance. Furthermore, uncovering these molecular identities will facilitate the discovery of human pathologies or phenotypic traits associated with specific enzymatic variants. Although our laboratory is actively pursuing the identification of these missing components, fully decoding the physiological relevance of this metabolic route remains a significant and compelling frontier for the field.

## Figures and Tables

**Table 1:** Summary of enzymes from the mammalian L-fucose degradation pathway.

Enzyme	Molecular Identity	Proof of Molecular Identity	Source Publications	Experiments for Definitive Validation
L-fucose dehydrogenase	HSD17B14	Structural similarity between human and bacterial enzymes; Co-purification with enzymatic activity from rabbit liver; Biochemical properties of recombinant rat, rabbit, and human enzymes (substrate specificity, kinetic parameters)	[48]	In vivo experiments: metabolic analysis of knockout mammalian cell lines and/or animal models
L-fuconolactonase	unknown	-	-	-
L-fuconate dehydratase	ENOSF1	Sequence and structural similarity between human and bacterial enzymes; Biochemical properties of the recombinant human enzyme (substrate specificity and kinetic parameters)	[50]	In vivo experiments: metabolic analysis of knockout mammalian cell lines and/or animal models
2-keto-3-deoxy-L-fuconate dehydrogenase	BDH2 (putative)	Sequence and structural similarity between human and bacterial enzymes; 2-Keto-3-deoxy-L-fuconate serves as an efficient substrate for the recombinant human enzyme	[47]	Biochemical studies (substrate specificity for structurally similar 2-keto-3-deoxy sugar acids); In vivo experiments: metabolic analysis of knockout mammalian cell lines and/or animal models
2,4-diketo-3-deoxy-L-fuconate hydrolase	unknown	-	-	-

## Data Availability

No new data were created or analyzed in this study. Data sharing is not applicable to this article.

## Abbreviations

The following abbreviations are used in this manuscript:

ORENZA	Orphan Enzyme Activities database
NAD(P)HX	endogenous damaged form of NAD(P)H
SETD3	actin-specific histidine methyltransferase
KEGG	Kyoto Encyclopedia of Genes and Genomes
GLUT1	glucose transporter type 1
SLC35C1	solute carrier family 35 member C1
EC number	Enzyme Commission number
ER	endoplasmic reticulum
FUTs	fucosyltransferases
GMDS	GDP-mannose 4,6-dehydratase
GFUS	GDP-L-fucose synthase
FCSK	L-fucose kinase
FPGT	fucose-1-phosphate guanylyltransferase
FucP	L-fucose:H+ symporter permease
FucU	Fucose mutarotase
FUOM	fucose mutarotase
FucK	L-fuculokinase
FucA	L-fuculose-1-phosphate aldolase
DHAP	dihydroxyacetone phosphate
FucO	lactaldehyde reductase
HSD17B14	hydroxysteroid 17-β-dehydrogenase 14
SDR	short-chain dehydrogenase/reductase superfamily
6-PGL	6-Phosphogluconolactonase
PPP	pentose phosphate pathway
G6PDH	glucose-6-phosphate dehydrogenase
SMP30	senescence marker protein 30
NAFLD	nonalcoholic fatty liver disease
NMR	Nuclear magnetic resonance
*fucD*	bacterial L-fucose dehydratase gene
FucD	bacterial L-fucose dehydratase
rTSβ	reverse thymidylate synthase
rTSγ	reverse thymidylate synthase
ENOSF1	Enolase Superfamily Member 1
rTSα	reverse thymidylate synthase
TYMS	thymidylate synthase
BDH2	4-oxo-L-proline reductase // type 2 (R)-β-hydroxybutyrate dehydrogenase
HEK293T	Human embryonic kidney 293
C785_RS20550	2,4-diketo-3-deoxy-L-fuconate hydrolase
LRA6 HEK293T	2,4-diketo-3-deoxy-L-rhamnonate hydrolase Human embryonic kidney 293
FAH	fumarylacetoacetate hydrolase family
FAHD1	fumarylacetoacetate hydrolase domain-containing protein 1
FAHD2A	fumarylacetoacetate hydrolase domain-containing protein 2A
FAHD2B	fumarylacetoacetate hydrolase domain-containing protein 2B
GWAS	Genome-Wide Association Studies
NHGRI-EBI	National Human Genome Research Institute-European Bioinformatics Institute
